# Coagulation and Fibrinolysis Biomarkers as Potential Indicators for the Diagnosis and Classification of Ovarian Hyperstimulation Syndrome

**DOI:** 10.3389/fmed.2021.720342

**Published:** 2021-08-25

**Authors:** Shuai Li, Yaqi Qian, Yue Pei, Kaiqi Wu, Shiming Lu

**Affiliations:** Department of Clinical Laboratory, Women's Hospital, School of Medicine, Zhejiang University, Hangzhou, China

**Keywords:** ovarian hyperstimulation syndrome, thrombin-antithrombin complex, plasmin alpha2-plasmin inhibitor complex, soluble thrombomodulin, tissue plasminogen activator-inhibitor complex, receiver operating characteristic

## Abstract

**Background:** Accurate diagnosis and classification of ovarian hyperstimulation syndrome (OHSS) is important for its management. We employed a new high-sensitivity chemiluminescence immunoassay to detect the thrombin-antithrombin complex (TAT), plasmin alpha2-plasmin inhibitor complex (PIC), soluble thrombomodulin (sTM), and tissue plasminogen activator-inhibitor complex (TPAI-C), and evaluated their diagnostic and classification performance for OHSS.

**Methods:** A total of 106 women were enrolled, including 51 patients with OHSS (25 mild or moderate OHSS, 26 severe OHSS), and 55 without OHSS (control group). TAT, PIC, sTM, and TPAI-C levels were measured using the Sysmex HISCL5000 automated analyzer.

**Results:** Compared to the control group, TAT, PIC, and TPAI-C levels were significantly higher (*P* < 0.001, *P* < 0.001, *P* < 0.001, respectively), whereas the sTM level was significantly lower (*P* < 0.001) in the patients with OHSS. The receiver operating characteristic was used to evaluate the diagnostic efficiency. For the diagnosis of OHSS, the area under the curves (AUCs) for TAT, PIC, sTM, and TPAI-C were 0.991, 0.973, 0.809, and 0.722, respectively. In particular, the sensitivity, specificity, positive predictive value, and negative predictive value for TAT and PIC were all above 90%. For the differential diagnosis of mild–moderate and severe OHSS, the AUCs for TAT, PIC, and TPAI-C were 0.736, 0.735, and 0.818, respectively. The cutoff values of TAT, PIC, and TPAI-C for the differential diagnosis of mild–moderate and severe OHSS were 11.5 ng/mL, 2.4 μg/mL, and 5.8 ng/mL, respectively. Based on these cutoff values, eight cases of mild–moderate OHSS exceeded the cutoff values, two of which developed to severe OHSS in the following days. However, of the remaining 17 cases of mild–moderate OHSS patients with negative biomarkers, none subsequently developed severe OHSS.

**Conclusions:** TAT, PIC, sTM, and TPAI-C can be used as sensitive biomarkers in the diagnosis of OHSS. Meanwhile, TAT, PIC, and TPAI-C also displayed remarkable potential in the classification of OHSS. In addition, the levels of TAT, PIC, and TPAI-C above the cutoff values in patients with mild–moderate OHSS might predict a high risk of developing severe OHSS.

## Introduction

Ovarian hyperstimulation syndrome (OHSS) is a major iatrogenic complication associated with controlled ovarian stimulation during *in vitro* fertilization (IVF). The reported incidence of OHSS varies markedly, and is estimated to be 0.5–5%, and even up to 10% in high-risk women (1–4). However, the true incidence of OHSS is difficult to estimate because of the lack of strict universally accepted diagnostic criteria. The precise pathogenesis of OHSS is unclear, but is believed to involve pro-inflammatory mediators produced by the use of human chorion gonadotrophin (hCG) for the triggering of final oocyte maturation (5). Women with OHSS demonstrate ovarian enlargement and increased vascular permeability. There is a shift of fluids from the intravascular compartment into the third space, mediated by the elevated levels of vascular endothelial growth factor (VEGF) secreted by the granulosa lutein cells (6).

Hypercoagulability is a common syndrome in patients with OHSS. Moreover, if hypercoagulability develops further, thrombosis may occur, which is the most serious and life-threatening complication of IVF. Thromboembolic disease has been reported in many sites of patients with OHSS, including in the internal jugular, subclavian, axillary, ulnar, popliteal, cortical, mesenteric, coronary, and cerebral vessels (7–9). To prevent the occurrence of thrombosis, it is crucial to accurately assess the hypercoagulability in patients with OHSS.

However, according to the existing diagnostic and classification criteria of OHSS, such as the Golan criteria, the guidelines published by the Practice Committee of the American Society for Reproductive Medicine and the Royal College of Obstetricians & Gynecologists, no hemostasis indicators are available (10–12). Currently, white blood cell (WBC) count and hematocrit (Hct) are the two most commonly used laboratory indicators. Unfortunately, an elevated WBC count may be secondary to a generalized stress reaction and hemoconcentration, and Hct mainly reflects intravascular volume depletion and blood viscosity (13). Neither can be used to reflect the hemostatic system. The absence of hemostasis indicators has resulted in a lack of laboratory guidance in the diagnosis and subsequent anticoagulant therapy of OHSS. Considering that conventional coagulation tests, such as activated partial thromboplastin time (APTT), prothrombin time (PT), and thrombin time (TT), are not suitable for assessing the hypercoagulability, novel coagulation and fibrinolysis biomarkers need to be explored.

The thrombin-antithrombin complex (TAT), plasmin alpha2-plasmin inhibitor complex (PIC), soluble thrombomodulin (sTM), and tissue plasminogen activator (t-PA)-inhibitor complex (TPAI-C) are four sensitive coagulation and fibrinolysis biomarkers in the hemostatic system. Elevated levels of these have been found in many underlying diseases. TAT, PIC, sTM, and TPAI-C levels are significantly higher in patients with disseminated intravascular coagulation (DIC) than in those with non-overt DIC, and TAT, sTM, and TPAI-C levels are significantly higher in patients with pre-DIC than in those with non-overt DIC (14). Yuying Cheng et al. confirmed the clinical value of the four biomarkers in predicting postoperative venous thromboembolism in total joint arthroplasty patients (15). In addition, the diagnostic and prognostic values of these biomarkers have also been demonstrated in patients with malignant tumors with venous thrombosis (16, 17).

In this study, we aimed to evaluate the diagnostic and classification value of TAT, PIC, sTM, and TPAI-C for OHSS, and preliminarily evaluate the potential use of these biomarkers in clinical practice in a Chinese population.

## Materials and Methods

### Participants

This study was performed at the Women's Hospital, School of Medicine, Zhejiang University (China) from July 2020 to February 2021. The study protocol was approved by the ethics committee of the hospital (approval number: IRB-20200133-R). Eligible participants were those who underwent IVF using either the gonadotropin-releasing hormone agonist (GnRHa) long protocol or the GnRH antagonist protocol and suffered from OHSS. The exclusion criteria were data missing from the database; age over 45 years; receiving GnRH agonist trigger or withholding hCG; any known hereditary or acquired thrombotic or bleeding disorder; having already received anticoagulant therapy; and chronic diseases, such as cardiovascular disease or diabetes mellitus.

During this period, 83 patients were diagnosed with OHSS, 32 of which were excluded based on the exclusion criteria, and 51 were enrolled in the final study, including 25 patients with mild or moderate OHSS and 26 with severe OHSS, according to Golan and Wasserman's 2009 criteria (10). All patients with OHSS were diagnosed within 9 days after oocyte retrieval. The control group consisted of 55 participants who underwent IVF using either the GnRHa long protocol or the GnRH antagonist protocol and did not develop OHSS during the same time period. Women in the control group were followed up throughout the first trimester to ensure that OHSS did not occur. The information collected for each participant was age; body mass index (BMI); IVF protocol; gonadotropin doses; antral follicle count; basal levels of follicle-stimulating hormone (FSH), luteinizing hormone (LH), estradiol, progesterone, and anti-Müllerian hormone (AMH); levels of estradiol on the day of hCG administration; levels of progesterone on the day of oocyte retrieval; and the number of oocytes retrieved.

### Controlled Ovarian Hyperstimulation Protocol

The standard treatment protocol for controlled ovarian hyperstimulation (COH) using the GnRHa long protocol or GnRH antagonist protocol was applied to all participants. In the GnRHa long protocol, GnRHa was administered in the midluteal phase preceding the cycle to downregulate estrogen production. Thereafter, COH was performed by the administration of both recombinant FSH (rFSH) and human menopausal gonadotropin (HMG), depending on age, BMI, antral follicle count, size and number of follicles, and estradiol levels. The initial doses of rFSH ranged from 150–225 IU/day, and those of HMG ranged from 75–150 IU/day. The dose was subsequently adjusted depending on the ovarian response, as evaluated by the E2 levels and combined with ultrasound. When at least three follicles reached a mean diameter of 17 mm, 6,500 IU of hCG was injected. The transvaginal ultrasound-guided oocyte retrieval was performed ~36 h after hCG injection.

In the GnRH antagonist protocol, rFSH or HMG was injected from the third day of the last menstrual period. The initial doses of rFSH and HMG were the same as those for the GnRHa long protocol, and the dose was subsequently adjusted, depending on the ovarian response. When the dominant follicle diameter was ~12–14 mm, the GnRH antagonist was administered until the day of hCG administration. When at least three follicles reached a mean diameter of 17 mm, 6,500 IU of hCG was injected, and oocyte retrieval was scheduled ~36 h after hCG injection.

### Blood Samples and Laboratory Assays

Blood samples were obtained immediately after OHSS was diagnosed and before any heparin treatment. For the control group, blood samples were obtained ~2 weeks after embryo transfer when the patient visited the hospital for the hCG test. If the hCG level was above 5.3 IU/L (reference interval: <5.3 IU/L), the participant would be followed up throughout the first trimester to ensure that OHSS did not occur, as previously described.

Blood samples were collected in a vacuum tube containing 0.109 M of dehydrated sodium citrate (BD Vacutainer Systems), centrifuged at 2,500 × *g* for 10 min, and then PT, APTT, thrombin time (TT), and fibrinogen concentration were measured by the clotting method on the STA-R MAX Coagulation Analyzer (Diagnostica Stago). The rest of the plasma samples were stored at −80°C for the assay of TAT, PIC, sTM, and TPAI-C. The four biomarkers were measured using the HISCL 5000 Automatic Chemiluminescence Immunoanalyzer (Sysmex Corporation) within 6 months. In addition, blood samples were collected in a vacuum tube containing EDTA-K2 (BD Vacutainer Systems) for the measurement of WBCs and Hct. WBC counts and Hct were determined on the Sysmex XN9000 Automatic Hematology Analyzer (Sysmex Corporation). All tests were performed with the original reagents and undertaken according to the manufacturer's instructions.

### Statistical Analysis

Statistical analyses were performed using the SPSS 20.0 software. A *P*-value < 0.05 was considered significant for all comparisons. The Shapiro-Wilk test was used to verify normality. Descriptive statistics are presented as mean ± SD for normally distributed variables and median (25th−75th percentile) for non-normally distributed variables. Parametric (*t*-test) and non-parametric (Mann-Whitney *U* test) analyses were performed for normally and non-normally distributed variables, respectively. Spearman's rank correlation analysis was used to analyze the association between the levels of these biomarkers and those of WBCs and Hct. Receiver operating characteristic (ROC) curves analysis was used to evaluate the diagnostic efficiency, and the maximum value of the Youden index served as the cutoff value. Sensitivities, specificities, positive predictive values (PPVs), and negative predictive values (NPVs) were calculated using standard formulas and were expressed as percentages [95% confidence interval (CI)].

## Results

### Baseline Clinical Characteristics of the Participants

Overall, 51 female patients with OHSS were enrolled in this study, including 25 with mild or moderate OHSS and 26 with severe OHSS. The control group consisted of 55 participants undergoing IVF who did not develop OHSS during the same time period. The baseline clinical characteristics of these participants are shown in Table 1. As expected, no significant differences were found in the conventional coagulation tests (PT, APTT, and TT) (*P* = 0.486, *P* = 0.286, *P* = 0.805, respectively). The levels of fibrinogen, WBCs, and Hct were significantly higher in the OHSS group than the control group (*P* < 0.001, *P* < 0.001, *P* = 0.001, respectively). The levels of TAT, PIC, sTM, and TPAI-C did not demonstrate any significant differences between the two therapeutic protocols (*P* = 0.946, *P* = 0.872, *P* = 0.467, *P* = 0.268, respectively).

**Table 1:** Baseline clinical characteristics of the participants.

	**OHSS**	**Controls**	***P***
	**(*n* = 51)**	**(*n* = 55)**	
Age (years)	30.3 ± 3.7	32.7 ± 4.8	0.007
BMI (kg/m^2^)	22.1 ± 2.7	21.9 ± 2.5	0.805
Antral follicle count	13.0 ± 5.9	11.6 ± 4.3	0.159
AMH (ng/mL)	7.02 (4.80–10.72)	2.37 (1.26–4.45)	0.000
Basal LH (IU/L)	5.93 (3.76–8.36)	4.81 (3.02–6.45)	0.093
Basal FSH (IU/L)	5.44 (4.64–6.24)	6.06 (4.80–7.55)	0.127
Basal estradiol (pmol/L)	129.99 ± 50.06	142.03 ± 72.51	0.320
Basal progesterone (nmol/L)	1.12 (0.63–1.49)	0.96 (0.77–1.26)	0.144
Estradiol on the day of hCG administration (pmol/L)	19,258.00 (12,155.00–31,990.00)	8,344.00 (4,445.00–11,404.00)	0.000
Progesterone on the oocyte retrieval day (nmol/L)	21.45 (13.33–43.02)	11.08 (8.36–17.13)	0.000
**IVF protocol [** ***n*** **(%)]**			
Long protocol	31 (60.7)	32 (58.2)	0.844
Antagonist protocol	20 (39.3)	23 (41.8)	0.844
Number of oocytes retrieved	18.0 ± 10.1	10.2 ± 6.4	0.000
**Conventional coagulation tests**			
PT (s)	12.9 ± 0.5	13.0 ± 0.6	0.486
APTT (s)	33.9 ± 3.6	34.6 ± 3.1	0.286
TT (s)	15.6 ± 1.1	15.7 ± 0.8	0.805
Fibrinogen (g/L)	5.04 ± 1.53	3.20 ± 0.57	0.000
**Blood routine examination**			
WBC (×109/L)	11.8 (9.2–14.9)	7.1 (6.4–9.6)	0.000
Hct (%)	41.8 ± 4.9	39.0 ± 2.5	0.001

*Data are presented as mean ± SD or median (25th−75th percentile) or No (%). OHSS, ovarian hyperstimulation syndrome; BMI, body mass index; AMH, anti-Müllerian hormone; LH, luteinizing hormone; FSH, follicle-stimulating hormone; hCG, human chorionic gonadotropin; IVF, in vitro fertilization; Gn, gonadotropin; PT, prothrombin time; APTT, activated partial thromboplastin time; TT, thrombin time; WBC, white blood cell; Hct, hematocrit*.

### Comparison of TAT, PIC, sTM, and TPAI-C Levels in Patients With OHSS and Control Patients

**Figure 1:**
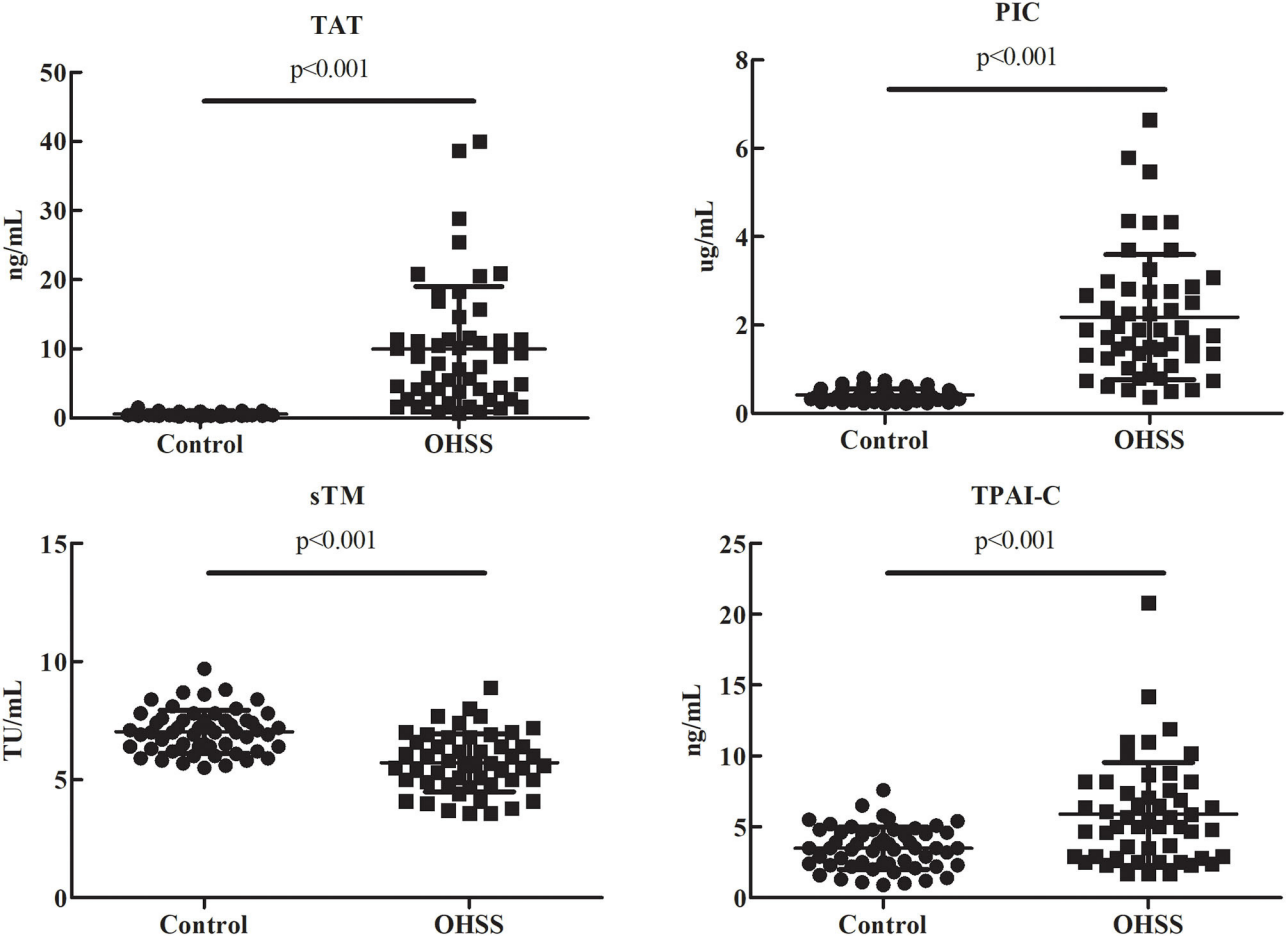
Comparisons of the thrombin-antithrombin complex, plasmin alpha2-plasmin inhibitor complex, soluble thrombomodulin, and tissue plasminogen activator-inhibitor complex levels in patients with ovarian hyperstimulation syndrome and control patients. TAT, thrombin-antithrombin complex; PIC, plasmin alpha2-plasmin inhibitor complex; sTM, soluble thrombomodulin; TPAI-C, tissue plasminogen activator-inhibitor complex; OHSS, ovarian hyperstimulation syndrome.

As shown in Figure 1, compared to the control group, TAT, PIC, and TPAI-C levels were significantly higher (*P* < 0.001, *P* < 0.001, and *P* < 0.001, respectively) and the sTM level was significantly lower (*P* < 0.001) in the OHSS group. The median plasma levels of TAT, PIC, sTM, and TPAI-C (OHSS vs. control group) were 7.4 (2.7–11.6) vs. 0.5 (0.4–0.6) ng/mL, 1.8 (1.2–2.8) vs. 0.4 (0.3–0.5) μg/mL, 5.6 (4.9–6.6) vs. 7.0 (6.4–7.5) TU/mL, and 5.1 (2.9–7.6) vs. 3.5 (2.3–4.6) ng/mL, respectively.

### Evaluation of the Correlations Between TAT, PIC, sTM, and TPAI-C Levels and WBC and Hct Levels

**Figure 2:**
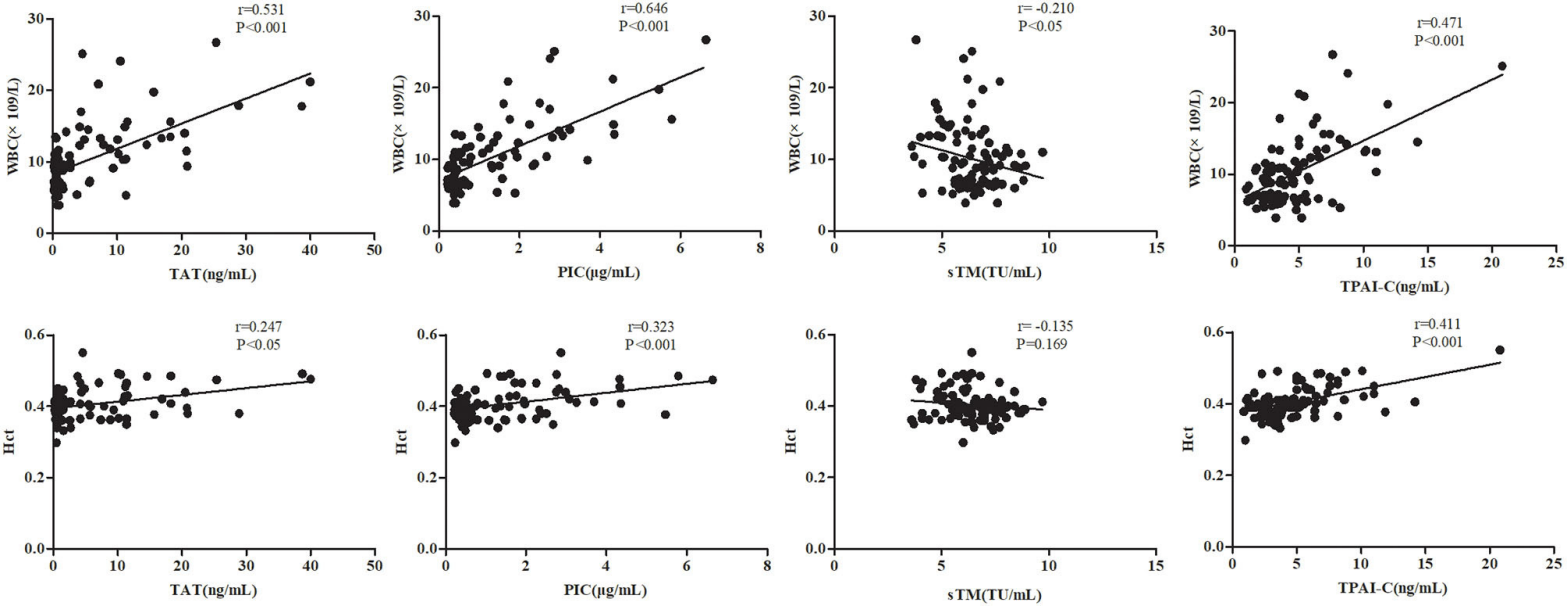
Correlations between the thrombin-antithrombin complex, plasmin alpha2-plasmin inhibitor complex, soluble thrombomodulin, and tissue plasminogen activator-inhibitor complex levels and white blood cell and hematocrit levels. TAT, thrombin-antithrombin complex; PIC, plasmin alpha2-plasmin inhibitor complex; sTM, soluble thrombomodulin; TPAI-C, tissue plasminogen activator-inhibitor complex; WBC, white blood cell; Hct, hematocrit.

As WBC and Hct levels are two routine laboratory indicators in the diagnosis and classification of OHSS, we investigated the correlations between TAT, PIC, sTM, and TPAI-C levels and WBC and Hct levels. As shown in Figure 2, TAT, PIC, and TPAI-C levels were positively correlated with WBC levels (*r* = 0.531, *P* < 0.001; *r* = 0.646, *P* < 0.001; *r* = 0.471, *P* < 0.001, respectively). However, the sTM level was negatively correlated with the WBC level (*r* = −0.210, *P* < 0.05). TAT, PIC, and TPAI-C levels were also positively correlated with the Hct level (*r* = 0.247, *P* < 0.05; *r* = 0.323, *P* < 0.001; *r* = 0.411, *P* < 0.001, respectively). There was no significant correlation between sTM and Hct (*r* = −0.135, *P* = 0.169). Based on a comparison, the correlations between the biomarker and WBC levels were higher than those between the biomarker and Hct levels. The correlations between sTM and WBC/Hct levels were lower than those between TAT/PIC/TPAI-C and WBC/Hct levels.

### Diagnostic Efficiency of TAT, PIC, sTM, and TPAI-C for OHSS

**Figure 3:**
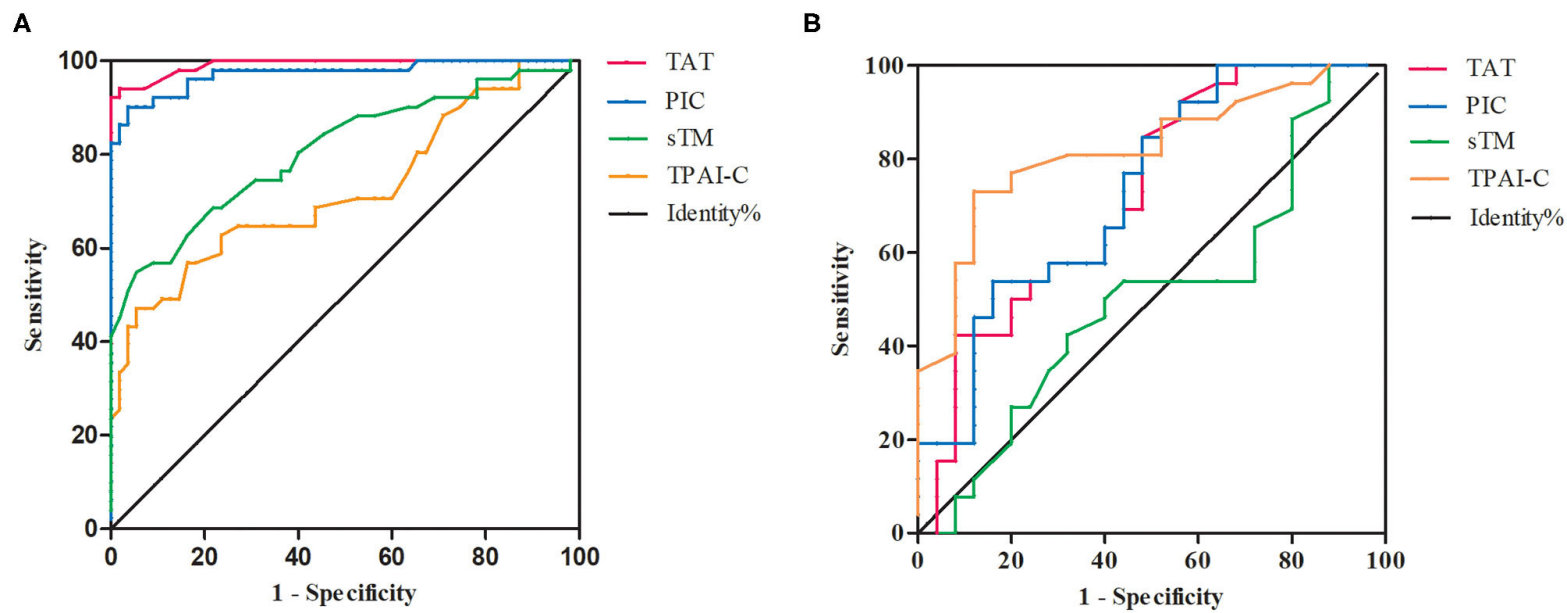
The receiver operating characteristic (ROC) curves of thrombin-antithrombin complex, plasmin alpha2-plasmin inhibitor complex, soluble thrombomodulin, and tissue plasminogen activator-inhibitor complex. **(A)** The ROC curves for the diagnosis of ovarian hyperstimulation syndrome (OHSS). **(B)** The ROC curves for the differential diagnosis of mild–moderate and severe OHSS. TAT, thrombin-antithrombin complex; PIC, plasmin alpha2-plasmin inhibitor complex; sTM, soluble thrombomodulin; TPAI-C, tissue plasminogen activator-inhibitor complex.

Given the marked changes in TAT, PIC, sTM, and TPAI-C levels in patients with OHSS, we further evaluated them as potential biomarkers for the diagnosis of OHSS using ROC curve analysis. Among the four biomarkers, the highest area under the curve (AUC) was observed for TAT and the lowest was observed for TPAI-C (Figure 3A). The optimal cutoff values were defined as the sum of maximum sensitivity and specificity. As shown in Table 2, the AUCs for TAT and PIC were 0.991 (95% CI, 0.980–1.000) and 0.973 (95% CI, 0.944–1.000), respectively, which were higher than the values for sTM and TPAI-C; namely, 0.809 (95% CI, 0.725–0.892) and 0.722 (95% CI, 0.622–0.820), respectively. In particular, the sensitivity, specificity, PPV, and NPV for TAT and PIC were all above 90%, which showed a good diagnostic value for OHSS. The specificities for sTM and TPAI-C were 94.5 and 83.6%, with a sensitivity of 54.9 and 56.9%, respectively. According to the ROC curve, the cutoff values of TAT, PIC, sTM, and TPAI-C for the diagnosis of OHSS were 1.2 ng/mL, 0.7 μg/mL, 5.7 TU/mL, and 5.0 ng/mL, respectively.

**Table 2:** Evaluation of thrombin-antithrombin complex, plasmin alpha2-plasmin inhibitor complex, soluble thrombomodulin, and tissue plasminogen activator-inhibitor complex efficiency [including the diagnostic efficiency for ovarian hyperstimulation syndrome (OHSS) and the differential diagnostic efficiency for mild–moderate and severe OHSS].

	**Diagnostic efficiency for OHSS**				**Differential diagnostic efficiency for severe OHSS**			
	**TAT**	**PIC**	**sTM**	**TPAI-C**	**TAT**	**PIC**	**sTM**	**TPAI-C**
AUC	0.991	0.973	0.809	0.722	0.736	0.735	0.506	0.818
95%CI	0.980–1.000	0.944–1.000	0.725–0.892	0.622–0.820	0.600–0.872	0.598–0.872	0.344–0.669	0.699–0.936
Cutoff value	1.2	0.7	5.7	5.0	11.5	2.4	6.1	5.8
Sensitivity (%)	94.1	90.2	54.9	56.9	42.3	53.8	42.3	73.1
Specificity (%)	98.2	96.4	94.5	83.6	92	84	68	88
PPV	97.9	95.8	90.6	76.3	84.6	76.5	48.9	86.4
NPV	94.7	91.3	69.3	68.7	68.4	61.8	44.4	75.9
Youden's index	0.923	0.865	0.494	0.405	0.343	0.378	0.103	0.611

*OHSS, ovarian hyperstimulation syndrome; TAT, thrombin-antithrombin complex; PIC, plasmin alpha2-plasmin inhibitor complex; sTM, soluble thrombomodulin; TPAI-C, tissue plasminogen activator-inhibitor complex; AUC, area under the curve; CI, confidence interval; PPV, positive predictive value; NPV, negative predictive value*.

### Differential Diagnostic Efficiency of TAT, PIC, sTM, and TPAI-C for Mild–Moderate and Severe OHSS

Compared to patients with mild–moderate OHSS, TAT, PIC, and TPAI-C levels were significantly higher in those with severe OHSS (*P* = 0.004, *P* = 0.004, *P* < 0.001, respectively), whereas the sTM level was not significantly different between the two groups (*P* = 0.940). We further investigated the differential diagnostic value of these biomarkers for mild–moderate and severe OHSS using ROC curve analysis (Figure 3B). As shown in Table 2, the AUCs for TAT, PIC, and TPAI-C in the differential diagnosis of mild–moderate and severe OHSS were 0.736, 0.735, and 0.818, respectively. However, the AUC for sTM was merely 0.506, which suggested that it cannot be used for the differential diagnosis of mild–moderate or severe OHSS. The cutoff values of TAT, PIC, and TPAI-C for the differential diagnosis of mild–moderate and severe OHSS were 11.5 ng/mL, 2.4 μg/mL, and 5.8 ng/mL, respectively. TPAI-C showed the highest differential diagnosis value, with a sensitivity of 73.1% and a specificity of 88%.

### Evaluations of the Outcomes of Patients With Mild–Moderate OHSS With Positive Biomarkers

The cutoff values of TAT, PIC, and TPAI-C for the differential diagnosis of mild–moderate and severe OHSS were 11.5 ng/mL, 2.4 μg/mL, and 5.8 ng/mL, respectively. Of the 25 patients with mild–moderate OHSS, eight had a positive result for at least one biomarker (above the cutoff values), and 17 had a negative result for all biomarkers (below the cutoff values). The management measures for these patients in clinical practice involved increasing fluid intake, supportive care, and intensive monitoring (blood pressure, pulse, daily weight, daily urine volume, laboratory parameters, etc.). Subsequently, we followed up these patients and found that two of the eight patients with positive biomarkers developed severe OHSS in the following days, and none of the 17 patients with negative biomarkers developed severe OHSS (Table 3).

**Table 3:** The outcomes of patients with mild–moderate ovarian hyperstimulation syndrome with positive biomarkers.

	**Clinical diagnosis**	**TAT, ng/mL**	**PIC, μg/mL**	**TPAI-C, ng/mL**	**Clinical outcomes**
Case 1	Mild OHSS	20.8^*^	1.2	2.4	Improved
Case 2	Moderate OHSS	20.0^*^	4.3^*^	5.0	Developed severe OHSS
Case 3	Mild OHSS	11.4	2.7^*^	2.9	Improved
Case 4	Moderate OHSS	2.7	3.7^*^	2.5	Improved
Case 5	Mild OHSS	2.6	3.5^*^	2.4	Improved
Case 6	Mild OHSS	6.9	1.4	6.5^*^	Improved
Case 7	Moderate OHSS	11.4	1.7	8.2^*^	Developed severe OHSS
Case 8	Mild OHSS	11.1	1.9	8.4^*^	Improved

* *Positive biomarkers: TAT > 11.5 ng/mL, PIC > 2.4 μg/mL, TPAI-C > 5.8 ng/mL. OHSS, ovarian hyperstimulation syndrome; TAT, thrombin-antithrombin complex; PIC, plasmin alpha2-plasmin inhibitor complex; TPAI-C, tissue plasminogen activator-inhibitor complex*.

## Discussion

Accurate diagnosis and classification of OHSS is important for the identification of suitable treatments. However, although a number of diagnostic and classification systems of OHSS have been developed, there are currently no universally agreed upon criteria (10, 11). Furthermore, current diagnostic and classification systems inadequately capture OHSS in clinical practice in a uniform manner, as no hemostatic indicators are involved. Hence, there is an urgent need to explore novel coagulation and fibrinolysis biomarkers for OHSS, despite considerable challenges.

In this study, we first investigated the levels of the conventional coagulation tests in patients with OHSS. No significant differences were found in the levels of PT, APTT, and TT between patients with OHSS and control patients. This might be because these tests are designed primarily to screen for coagulation factor deficiencies, rather than hypercoagulability, which is usually related to excessive coagulation factors (18, 19). Although fibrinogen levels were significantly higher in patients with OHSS vs. control patients, however, given that fibrinogen was an indicator of acute phase of reaction, and the increase of fibrinogen levels came not only from disorders of the hemostasis system, but also from inflammation and other stress responses, fibrinogen was not a specific indicator for the diagnosis and classification of OHSS.

Our results indicated that TAT, PIC, sTM, and TPAI-C had good diagnostic performance for OHSS; meanwhile, we showed that TAT, PIC, and TPAI-C can also be used to classify the severity of OHSS. TAT is a molecular complex composed of thrombin and antithrombin and is considered to be a sensitive marker of thrombin formation and coagulation activation. PIC is a molecular complex composed of plasmin and alpha2-plasmin inhibitor, and is a marker of plasmin formation and fibrinolysis activation. sTM is not only an indicator of endothelial injury, but can also be combined with thrombin to play an anticoagulant role. TPAI-C is formed through the combination of t-PA and plasminogen activator inhibitor 1, and is a marker of endothelial injury and fibrinolysis activation. Therefore, these biomarkers are highly sensitive coagulation and fibrinolysis indicators and may highlight even minimal hemostatic activation (14). The data of the present study also supported this. We found that TAT, PIC, and TPAI-C levels were significantly higher, whereas sTM levels were significantly lower, in patients with OHSS vs. control patients. This suggested that there was an imbalance between coagulation and fibrinolysis in patients with OHSS. Although the exact mechanism has not yet been fully elucidated, the imbalance may play a vital role in the thrombosis of patients with OHSS. These results are in accordance with those of several previous studies (20, 21). In addition, in contrast to the other biomarkers, sTM levels in patients with OHSS were significantly lower than those in control subjects. As sTM is not only an indicator of endothelial injury, but also combines with thrombin to play an anticoagulant role, this decrease in levels may contribute to the tendency of thrombosis development in patients with OHSS.

As WBC and Hct levels are the two most commonly used indicators in the existing diagnostic and classification criteria of OHSS, we investigated the correlations between biomarker levels and WBC and Hct levels. Except for sTM, significant correlations were observed between the biomarker levels and WBC and Hct levels. The highest correlation was observed between PIC and WBC levels (*r* = 0.646, *P* < 0.001). Owing to the significant correlations between the biomarkers and WBC levels, Hct levels further confirmed their diagnostic and classification value. The correlations between the biomarker and Hct levels were relatively weak. As Hct mainly reflects hemoconcentration, the relatively weak correlation implies that other mechanisms contribute to the hypercoagulability in OHSS other than hemoconcentration. This is consistent with the results of a study by Zohav et al. (22).

The AUCs for the diagnostic biomarkers TAT, PIC, sTM, and TPAI-C were 0.991, 0.973, 0.809, and 0.722, respectively. These biomarkers showed good diagnostic efficiency for OHSS. In particular, the sensitivity, specificity, PPV, and NPV for TAT and PIC were all above 90%, which showed extremely high diagnostic value. Once a diagnosis of OHSS is made, disease severity should be classified. The treatment approach for the clinical management of OHSS is multifaceted and individualized based on disease severity and progression. Most mild and moderate cases of OHSS are self-limited and require only intensive monitoring in the outpatient department; however, severe OHSS requires hospitalization to relieve symptoms and control the progression of the disease (23, 24). In this study, TAT, PIC, and sTM levels in the mild–moderate OHSS group were significantly higher than those in the control group (*P* < 0.001, *P* < 0.001, and *P* < 0.001, respectively). Meanwhile, TAT, PIC, and TPAI-C levels in the severe OHSS group were significantly higher than those in the mild–moderate OHSS group (*P* = 0.004, *P* = 0.004, and *P* < 0.001, respectively). This revealed that the hypercoagulability in OHSS was a gradual process. Therefore, these biomarkers might have important roles in its classification. The ROC curve analysis results of the present study also supported this. We investigated the differential diagnostic value of these biomarkers for mild–moderate and severe OHSS. Except for sTM, all biomarkers showed significant potential value for the classification of OHSS. The highest AUC was observed for TPAI-C (0.818), with a sensitivity of 73.1% and a specificity of 88%, which suggested that TPAI-C was the most optimal biomarker. The cutoff value for TPAI-C in the differential diagnosis for mild–moderate and severe OHSS was 5.8 ng/mL.

In this study, to further validate the value of these biomarkers in clinical practice, we followed up the outcomes of the patients with mild–moderate OHSS. There were eight patients with mild–moderate OHSS who exceeded the cutoff values obtained by ROC analysis, two of which developed severe OHSS in the following days. However, of the remaining 17 patients with mild–moderate OHSS with negative biomarkers (below the cutoff values), none subsequently developed severe OHSS. These results indicated that the patients with mild–moderate OHSS with positive biomarkers were at a high risk of developing severe OHSS; namely, the presence of positive biomarkers in patients with mild–moderate OHSS might predict a poor prognosis. Considering that the treatment approach for OHSS is multifaceted and individualized based on disease severity and progression, these biomarkers could help identify high risk patients with mild–moderate OHSS who need to be more closely monitored and who may even require early prophylactic anticoagulant therapy.

In addition, the clinical application of these biomarkers has been markedly restricted owing to the inconvenient and inefficient detection methodology used in the past. Currently, with the development of the high-sensitivity chemiluminescence immunoassay, these biomarkers can be detected quickly and automatically in a clinical laboratory. For the test used in this study, the minimum volume for a sample was 20 μL, and the results were available within 17 min. The improvement of the methodology will facilitate the spread of these biomarkers to thousands of laboratories in China.

There were some limitations in this study. The sample size was not sufficiently large, especially after grouping. Therefore, larger sample studies are needed. Another limitation is that all participants were subjected to a single sampling. If a series of sampling is performed at different time points, such as during COH, on the day of hCG administration, and on the oocyte retrieval day, this will allow the evaluation of the value of these biomarkers more comprehensively, which may be the focus of our future studies.

In conclusion, we found that TAT, PIC, sTM, and TPAI-C can be used as sensitive biomarkers in the diagnosis of OHSS. Meanwhile, TAT, PIC, and TPAI-C also displayed remarkable potential in the classification of OHSS. In addition, the levels of TAT, PIC, and TPAI-C above the cutoff values in patients with mild–moderate OHSS might predict a high risk of developing severe OHSS.

## Data Availability Statement

The raw data supporting the conclusions of this article will be made available by the authors, without undue reservation.

## Ethics Statement

The studies involving human participants were reviewed and approved by the ethics committee of the Women's Hospital, School of Medicine, Zhejiang University (China). Written informed consent for participation was not required for this study in accordance with the national legislation and the institutional requirements.

## Author Contributions

SLi, YQ, and SLu conceived the idea and designed the study and drafted the article. SLu revised the article. YP and KW contributed the collection and analysis of the data. All authors have approved the submitted version.

## Conflict of Interest

The authors declare that the research was conducted in the absence of any commercial or financial relationships that could be construed as a potential conflict of interest.

## Publisher's Note

All claims expressed in this article are solely those of the authors and do not necessarily represent those of their affiliated organizations, or those of the publisher, the editors and the reviewers. Any product that may be evaluated in this article, or claim that may be made by its manufacturer, is not guaranteed or endorsed by the publisher.
